# Pepsin

**Published:** 1874-04

**Authors:** R. T. Edes


### ARTICLE VI.

### *Pepsin.*

BY R. T. EDES, M. D.

Read before the Dorchester Medical Club.

Pepsin is the peculiar ferment present in the gastric juice. As a physiological experiment, the juice itself can be used for artifical digestion, but obviously cannot be obtained in sufficient quantity for therapeutic application. Various methods have been employed to obtain the active principle in a form suitable for medical use. Boudault's, Morson's, Beal's and various others have been prepared in Europe, but it is sufficient to mention only the three first, as the others have not come into use here. " The pepsin of commerce is either mucus of the stomach scraped off and dried, or a mixture of pepsin, peptones and starch, containing a little lactic acid."* Beale's corresponds to the first part of the description. Boudault's and Morson's to the second.

* Gmelin's Handbook of Chemistry. 1870.

By far the larger part of these consists of starch, as a diluent is necessary to enable them to be dispensed.

The process of Mr. Scheffer, of Louisville, consists in macerating the chopped mucus membrane of the hog's stomach in acidulated water, and, after allowing the mucus to settle, precipitating the pepsin by a saturated solution of common salt.

The precipitate is dried upon a cloth, its digestive strength estimated by experiment, and then mixed with sugar of milk in such proportion that 10 grains dissolves 120 grains of hard-boiled white of egg in four to six hours under the appropriate conditions.

Although this experiment cannot be considered as exactly representing the amount of work done in the stomach, yet it furnishes the only available means of comparison between different prepartions. I have examined, in this way, Boudault's neutral, Morson's, Procter's, Hawley's, and the "Aromatic Liquid Pepsin," which has been extensively advertised within a few weeks. Procter's and Hawley's, the first of which is avowedly, and the second probably, made by Scheffer's process, were exceedingly efficient, leaving but a small residue, and that pulpy and friable. The action of Morson's and of Boudault's was very slight, in fact hardly perceptible. and the same is true of the "Aromatic Liquid Pepsin."† These experiments were performed only twice with Boudault's and Morson's, with the same result, since there seems to be no reason for using imported preparations, which are more expensive, less elegant, and vastly less efficient than those which we have furnished us here. As regards the "Aromatic Liquids," the experiment was repeated twice more with a similar result.

I did not succeed in making Procter's, which was the preparation used in subsequent experiments, dissolve the full amount of albumen, but it may easily be imagined that

† Since writing this sentence, I have noticed in the Practitioner an examination of three English preparations, Savory & Moore's, Morson's pepsina porci, and Squire's. The digestive power of all was extremely slight.

the variation in the amount of heat, in the degree of comminution, and especially in the amount of stirring and of shaking, might have a great influence on the quantitative result. In each experiment, however, the different preparations were treated in a precisely similar manner in these respects. At first, the residue was weighed to determine the amount of albumen dissolved; but owing to the differences in the amount of water absorbed, and the difficulty in properly drying the residue, especially when partly digested, I think that the appearance of the fragments of albumen is, for practical purposes, quite as reliable.

It is very well known that many manufacturers, being aware that sundry other drugs have their uses in the treatment of dyspepsia, but forgetting that, in physiology and therapeutics, two and two do not always make four, have combined pepsin with any three or four of these drugs in the much advertised and, I fear, much used compound aromatic fragrant tonic and invigorating elixirs of pepsin, bismuth, strychnia, quinia, cod-liver oil, lacto-phosphate of lime, and Liebig's extract of beef.

Mr. Scheffer, whose experiments upon pepsin have extended much beyond the preparation of the most reliable form of this useful agent, has shown not only that the pepsin is usually precipitated by the alkalinity of the fluid used to hold the bismuth in solution, but that the bismuth itself precipitates the pepsin. The action of an elixir of bismuth, strychnia and pepsin is shown in this bottle, which may be called a visible dyspepsia, the pieces of albumen being shrunken, dark and tough, without the faintest trace of solution. I also tried a granular effervescent preparation of the same kind, with absolutely no digestion. Finally, I added the ordinary subnitrate of bismuth to a bottle containing the usual materials, with the result which you see, (no digestion.)

I think we may fairly conclude that any preparation purporting to contain both bismuth and pepsin contains *none* of the latter; and also the bismuth and pepsin in the form of powder is not an eligible combination. If it is desired to give both they will be more efficient separately.

As regards alcohol, Mr. Scheffer stated, in one of his earlier essays, that "dry pepsin, precipitated with alcohol from its solution, did not act at all on albumen." This he afterwards found to be a mistake, since it was not from an acid solution that he obtained a precipitate by alcohol, but from one rendered neutral by carbonate of soda. The carbonate of soda permanently modifies the digestive properties of pepsin, and destroys its action on freshly-coagulated albumen. It still acts, in a different manner, upon partly digested albumen. He showed, however, and this phial shows the same thing, that alcohol does not, in small quantity, prevent the digestive process, but retards it. You notice that the amount of albumen remaining, although evidently acted upon, is much larger than that in the standard phial. It would seem, then, that a wine of pepsin may contain some of the ferment, although, from the activity of the mucous membrane, after the wine has been prepared from it, the quantity extracted cannot have been large. Another property of pepsin, which I have not before mentioned, indicates that it is to be found in rennet wine or liquid rennet. This is used for the purpose of coagulating milk, which it will do at ordinary temperatures. This coagulation is not due to its acid, as we found by comparing its action with that of dilute muriatic acid of about the same strength. If to the tube containing milk unacted upon by the acid, pepsin was added, coagulation then took place.

Also, if the rennet wine were *boiled*, it lost its action upon milk; so that, so far as its power in coagulating milk goes, rennet wine behaves like an acid solution of pepsin. The proof, however, must be in the direct experiment, the result of which you see. (A small amount of digestion. The temperature was a few degrees lower than in previous experiments, and the digestion of the albumen in pepsin, made at the same temperature for comparison, also took place slowly.)

I have tried no preparation of the wine of pepsin. There seems no reason why these should differ from the liquid rennet.

It has been thus far assumed that the conditions in these experimental phials and in the human stomach were nearly the same. It is fair, however, to state certain points of difference. In the stomach, the agitation and mixture of the solvent and food is, or ought to be, much more thorough. In the stomach, the portion digested is absorbed nearly as fast as it is formed.

In the stomach, certain incompatibles may be removed before the action of the pepsin is compelled. This is chiefly true as regards alcohol, which, in ordinary doses, is probably absorbed long before the time for the digestion of an ordinary meal is over. It is not infrequently objected that the amount of albumen dissolved by pepsin is entirely insignificant in proportion to the amount usually taken at a meal, even by and invalid. This is true, if we consider only the amount dissolved, with a given amount of acid, in these experiments, which are intended to represent only the relative value of different varieties. Mr. Scheffer has, however, shown that, under conditions resembling more closely those of stomach digestion, pepsin may be made to dissolve much more. By the successive addition of acid, water and albumen, the amount of the latter dissolved can be very much increased, so that half a grain of purified pepsin, that is, the pepsin before the addition of the sugar of milk, dissolved 1500 grains of albumen, and perhaps would have done more.

In another experiment, he found that, upon adding a saturated solution of common salt to clear fluid obtained by the digestion of albumen, a precipitate was produced which, in its turn, being dissolved in acidulated water, digested still more albumen, and, by continuing this process, twenty grains of saccharated pepsin dissolved at the rate of between 4000 and 5000 grains. That is, we see that pepsin is a true ferment, and not a chemical reagent; so that although the amount of albumen digested in the test tube in a given time furnishes a fair test of the value of the preparation, it does not limit its activity in an organ which absorbs more or less of formed products, and which, for all we know, may be capable of rapidly returning to its own interior the ferment which, while digesting other albuminoids and giving to them in their turn catalytic power, does not lose its own activity.

*Conclusions.*—Much of the dissatisfaction with pepsin expressed by physicians is due to the use of preparations which contain little or none of it.

The pepsin made by Scheffer's process is by far superior to any other in ordinary use.

The wine is feeble, but not necessarily inert.

Elixirs of pepsine and bismuth are humbugs.

Pepsin should be administered with an acid, and with as few drugs as possible. A small amount of alcohol is not inadmissible, but a large amount retards digestion.

Its beneficial action is *not limited* by the amount of albumen which it dissolves in a test tube without change or renewal of any of the contents.—*Boston Med. and Surg. Journal.*

